# Anti oxidative/neuro-inflammation properties of *Withania somnifera root extract* on rotenone induced stress in rat brain

**DOI:** 10.6026/97320630019729

**Published:** 2023-06-30

**Authors:** Vishala Epuri, Lavanya Prathap, Venkateshwar Reddy, Madhan Krishnan

**Affiliations:** 1 Research Scholar, Saveetha Institute of Medical and Technical Sciences, Chennai- 600077, Tamil Nadu, India; 2 Department of anatomy, SVS medical college, Mahaboob Nagar, Yenugonda- 509001, Telangana, India; 3 Department of Anatomy, Saveetha Dental College and Hospitals, Saveetha Institute of Medical and Technical Sciences, Velappanchavadi, Chennai- 600077, Tamil Nadu, India; 4 Chettinad Hospital and Research Institute, Chettinad Academy of Research and Education, Kelambakkam- 603103, Tamilnadu, India

**Keywords:** Parkinson's disease (PD), *Withania somnifera* (WS), lipid peroxidation (lpo), superoxide dismutase (sod), catalase (cat), microglia, neurodegeneration.

## Abstract

Parkinson's disease (PD) is a neurological condition that worsens with age (i.e., 1% of people over 65) with no permanent cure. Hence, finding a disease-modifying agent with fewer undesirable side effects is urgently needed. Parkinson's disease (PD) pathology results in the degeneration of dopaminergic (DAergic) neurons by accumulating lewy bodies, alpha-synuclein (-syn), lowering anti-oxidants, increasing neuronal inflammation, and altering neuron shape. A well-researched natural substance called *Withania somnifera* (WS) has a potent anti-oxidative, anti-inflammatory, and anti-neurodegenerative impact. WS, sometimes called as Indian Ginseng, is a subtropical undershrub of the Solanaceae family together with Ashwagandha. In the current work, EWSR's anti-inflammatory and neuroprotective efficacy was assessed in relation to rotenone-induced oxidative stress (i.e., LPO, CAT, and SOD and GSH), microglial activation, and neurodegeneration in the rotenone rat PD model. In ROT-induced brains, EWSR therapy resulted in a considerable decrease in LPO and increased levels of the antioxidants SOD, CAT, and GSH. Furthermore, our research showed that the intraperitoneal treatment of EWSR (40 mg/kg) in rotenone-induced rats reduced microglial activation and neuron loss in the substantia nigra (SN) and hippocampus caused by rotenone-induced neurotoxicity. Based on the observations, EWSR can be considered as an excellent source for neuroprotection, due to its significant anti-oxidative, anti-inflammatory, anti-neurodegenerative and anti-microglial properties when administered individually and in combination with known anti-inflammatory compounds (Doxycycline and Ellagic acids). But, further research is required before replacing the known neuroprotective treatments with phytochemical treatments.

## Background:

Parkinson's disease is a neurodegenerative condition characterized by the progressive degeneration of dopamine (DA) neurons in the substantia nigra pars compacta (SNc) and the loss of dopaminergic nerve terminal fibers in the striatum. This disorder commonly occurs as individuals' age. Dopaminergic dementia leads to a sharp decline in the amount of the DA neurotransmitter, which regulates movement, in the striatum. As a result, dopaminergic neurodegeneration leads to stiffness, aberrant gait patterns, and mobility issues [1]. Even though the exact aetiology of dopaminergic neuronal death in Parkinson's disease (PD) is unknown, current research shows that oxidative stress and neuroinflammation play significant roles in the pathogenesis of PD [2].In agriculture, rotenone (ROT), an inhibitor of mitochondrial complex I, is frequently used as an herbicide and pesticide. Due to its complex I inhibitory property, an ROT challenge causes pathological traits in animals that are comparable to those found in PD patients. As a result, animals who have received ROT treatment are a potential animal model with construct validity [3]. The majority of the clinical characteristics seen in the pathophysiology of human PD are reproduced by the ROT model, including the loss of DA neurons in the substantia nigra pars compact (SNc) and increased oxidative stress and neuroinflammation in the nigrostriatal dopaminergic pathway [4]. Additionally, an ROT challenge to animals resulted in the accumulation of nigral iron, Lewy pathology, DJ-1 acidification and translocation, proteasomal failure, and the production of -synuclein cytoplasmic inclusions in DA neurons [1, 5]. As a result, the rat model of ROT treatment is a good choice for researching potential new treatments for Parkinson's disease (PD) that target neuroinflammation and oxidative stress [6, 7 8]. The PD symptoms are reduced by the medications that are now on the market, but they cannot stop the disease's progression. In last 2-3 decades the researchers focus has been shifted to phytobased therapy i.e., essential oils, extracts derived from medicinal plants, microbial metabolites, trace elements with beneficial biological effects including anticonvulsant [9], analgesic [10,11], anxiolytic [12], antidepressant [13], antioxidant [10], and anti-inflammatory. Doxycycline (i.e., antibiotic tetracyclic) exhibits neuroprotective effects that were previously thought to be related to its anti-inflammatory properties and prevention of amyloid aggregation; however, a new mechanism by which it may exert its neuroprotective properties has been proposed i.e., reducing the formation of reactive oxygen species originating from mitochondria and preventing the aggregation and seeding of recombinant alpha-synuclein (aSyn) [14]. In a rat model of Parkinson's disease, dopaminergic function is reported to be restored by doxycycline, initiating a neuroprotective pathway. Doxycycline therapy may be an effective way to treat synucleinopathies, however there aren't many strong arguments in favour of this [15]. It has been found that ellagic acid (EA), a polyphenol and naturally occurring dimeric derivative of gallic acid, can be found in a variety of fruits and nuts, including grapes, strawberries, raspberries, pomegranates, and walnuts. This polyphenol works as an antioxidant and anti-inflammatory in mammalian cells [16, 17]. EA has been demonstrated to have anticholinesterase and antioxidant activities in in vitro experiments. EA's ability to protect neurons has been demonstrated in a number of experimental models [18]. Additionally, EA can improve cholinergic transmission, which contributes to its neuroprotective properties, and reduce cognitive disruption following scopolamine injection, prevent cognitive deficits brought on by traumatic brain injury, and recover memory loss in a 6-hydroxydopamine-induced PD model in a rat model, where A-25-35 was injected intra hippocampally [19]. The Indian Ayurvedic system of medicine holds *Withania somnifera* (Ashwagandha) in high regard. In particular, it is utilised as a nervine tonic and is beneficial for treating a variety of illness conditions. Withaferin A, with a one, and other flavonoids with potent antioxidant capabilities are only a few of the active ingredients found in *Withania somnifera* (Ws), which also contains a variety of other substances [20]. Numerous scientific investigations on Ws have been conducted in the past, demonstrating its anti-oxidative properties, synergistic effects with other therapeutic herbs, effectiveness in raising catecholamine levels, and control of apoptotic processes. According to Bhattacharya *et al*., rats' cortical and striatal antioxidant enzyme activity (SOD, CAT, and Glutathione peroxidase) are changed by glycol with a nolides the active components of Ws [13]. In the current work, EWSR's anti-inflammatory activity will be assessed in relation to rotenone-induced oxidative stress, microglial activation, and neurodegeneration as well as its neuroprotective efficacy in the rotenone rat PD model.

## Material and methods:

Ethanolic *Withania somnifera* root extract (EWSR) -dried roots of *Withania somnifera* was bought from local markets of Hyderabad, Telangana state. The roots were washed thoroughly with distilled water, room dried, powdered and soaked in 50% ethanol (Absolute ethanol diluted with double distilled water) and left for continuous mixing on magnetic stirrer at room temperature for next 7 days. The extract was later on filtered fine sieves and Whatman's No1 filter paper and later lyophilized into powder.

## Experiment design:

Experimental planning The National Institute of Nutrition (NIN), Hyderabad, India, provided 36 male Wistar rats weighing 160-200 grammes. Prior to being housed in separate polypropylene cages, the rats were maintained in quarantine. They were fed a regular pellet diet (NIN, Hyderabad) during the trial, and water was available at all times. Six rats each made up each of the four groups of rats. Sunflower oil was given to the first group (controls), rotenone (ROT) dissolved in sunflower oil (2 mg/kg bw) was given daily to the second through sixth groups via intraperitoneal route for 4 weeks, and the seventh through ninth groups also received individual treatment with EWSR (100 mg/kg bw), Doxycycline (Doxy, 40 mg/kg bw), and Elagic acid (EA, 40 mg/kg bw) for 5-6 weeks via gavage. After Parkinson's disease symptoms were confirmed, the rats were kept for 70 days, from the first day of neurotoxic induction to the 28thday, during which time neuroprotectants were administered for the following 5 weeks, followed by 1 week of observation after neuroprotective treatments. Rat were killed via cervical dislocation, and their brains were removed and immediately placed in extremely cold temperatures (starting at 4°C and subsequently dropping to 20°C) for further research.

##  Pro and anti-oxidative markers:

## Lipid Peroxidation (LPO):

Lipid peroxidation is quantified by the measurement of secondary products, such a Malondialdehyde (MDA) using the spectroscopic method. The optimized procedure involves protein precipitation with trichloroacetic acid, by acid hydrolysis form the MDA thiobarbituric acid complex (MDA-TBA). The intensity of lipid peroxidation in terms of formation of TBA reactive substances was examined by the modified technique of Ester Bauer and Cheeseman. The homogenates including 0.5 mg protein were mixed with 0.5 mL of TCA (20%) + 1 mL of TBA (0.67%) and incubated for1h at 100°C. After cooling, samples were centrifuged, and the supernatant was collected. The absorbance of reaction mixtures was measured at 535 nm wavelength using a blank containing all the reagents except for homogenates [21].

## Superoxide Dismutase (SOD):

SOD activity in the tissue was assessed indirectly based on the enzyme's capacity to prevent O2 dependent pyrogallol autooxidation. The assay system in a final volume of 1.0mL consisted of 600µL of 83.3mM Tris-HCl buffer (pH 8.2), 100µL of 0.5mM DETPA, 50µLenzyme (supernatant), 50µLof Tris-EDTA, 50µL of 0.01N HCl, 100 µLof H2O2 mix well then initiate the reaction by the adding the 50µL of 3.97mM Pyrogallol. Increase absorbance read at 420nm with suitable blanks was recorded spectrophotometrically. The values are expressed in Units/min/100mg protein. 10% homogenate prepared in Tris-HCl (pH 8.2) centrifuged at 13,000rpm, supernatant used for the assay of SOD [22].

## Catalase:

Catalase was measured spectrophotometrically using modified Bonaventura *et al*. 1972 [23] procedures. 50µL of the supernatant were added to the reaction mixture, which also contains 2 mL of phosphate buffer (pH 7.0) and 1 mL of 30 mM H2O2. At 240 nm, catalase activity was measured. The catalase activity was assessed using the 43.6M cm-1 molar extinction coefficient of H2O2. When expressed as units/mg of protein, activity is defined as 1 mmol of H2O2 destroyed per minute [6].

## Reduced Glutathione (GSH):

The concentration of GSH (µM/g wet tissues) in brain tissues was estimated by evaluating free-SH groups, using 5,5'-dithiobis (2-nitrobenzoic acid) (DTNB) as a substrate. The yellow color developed was read immediately at 412 nm and expressed as µmol GSH/g tissue. The protocol was based on the modified method of Sedlak and Lindsay [8].

## Histopathology studies:

## Hematoxylin and Eosin staining (Hippocampus and Midbrain):

For one to two days, brain tissues were preserved in 10% formaldehyde. The tissues were successively hydrated for 10-15 minutes with distilled water and alcohol concentrations of 100%, 75%, 50%, and 30%. Different alcohol concentrations were employed to dehydrate the tissue, including 30%, 50%, 70%, 90%, and 100% alcohol for 20 minutes at a time, followed by two changes of xylene for 10 minutes, before the tissue was embedded in wax at a temperature of 55°C (minimum two changes required). Using a rotary microtome, embedded tissue slices of 5-7 microns were created (Leica RM2255 Fully Automated Rotary Microtome). The brain sections were examined with an Olympus microscope after being stained with hematoxylin and eosin [24].

## Golgi-cox staining technique:

The Golgi-Cox staining solution was made by combining 200 mL of distilled water (dw) with 80 mL of 5% potassium chromate (dissolved in cold dw), 100 mL of 5% mercuric chloride (dissolved by adding it slowly in hot dw), and 100 mL of 5% potassium dichromate (dissolved in warm dw). The diluted potassium chromate solution was slowly and constantly stirred into the potassium dichromate-mercuric chloride combination. After being prepared, the solution was kept in a dark, acid-free bottle for three to seven days before being filtered and applied to the stain [25].

## Procedure:

The brains were immediately placed in Golgi-COX solution after being dissected (i.e., the solution 50 times corresponds to tissue volume). The brains were stored in a dark environment for 3-7 days before being moved into a 30% sucrose solution and then sectioned. 5-micron sections of the brain were cut using a vibratome's sectioning chamber (NVSLM1, WPI), which was filled with a 6% sucrose solution. After rinsing in DW for one minute, the brain slices were placed in ammonium hydroxide or ammonia solution (1, 3 or 2,3 of each) and incubated for 5-7 minutes. They were then rinsed in DW for another minute. The sections were then placed into a solution containing 0.5% sodium thiosulfate and 0.2% sodium metabisulfite, incubated for 7 to 10 minutes, and then washed in DW for 5 minutes. The sections were then mounted with DPX and put onto 2% gelatin-coated slides, where they were dehydrated by dipping them in isopropyl alcohol.

## Microglia Staining (Lectin immunohistochemistry):

For one to two days, the brain was preserved in chilled PBS buffered paraformaldehyde. The vibro slicer was used to create 10-micron brain sections (NVSLM1, WPI), which were then twice washed in distilled water for two minutes. To raise the surface tension, the sections were allowed to soak in 0.1% Tween-20 in PBS for 20 minutes. To avoid non-specific staining, the sections were first incubated in 1% BSA for 30 minutes after being blocked with 3% H2O2 in PBS for 10 minutes. The sections were rinsed three times for two minutes with PBS Tween-20 before being treated with the primary antibody and 10 g/mL biotinylated lectins (RCA120, Lectin from *Ricinuscommunis* agglutinin (caster been) Sigma chemicals-L7886), GSA-B4 (BSA-B4) (Isolectin B4 Biotin-labeled from *Bandeiraeasimplicifolia (Griffoniasimplicifilia))* Sigma chemicals-L2140) overnight at 4°C [26].

Sections were washed with PBS Tween-20 three times for two minutes after an overnight incubation. Extra Avidin Peroxidase (HRP, Sigma Chemicals-E28826) was incubated with the sections for 2 hours at room temperature in order to identify the lectins. Following a three-by-two-minute PBS Tween-20 rinse, the sections were coloured in 5 to 10 minutes using DAB (3, 3'-Diaminobenzidine tetrahydrochloride hydrate, D5637, Sigma chemicals). Sections were transferred to gelatin-coated slides and then ethanol-dehydrated. It is mounted with DPX and xylene-cleared. To perform controls, RCA-I was incubated with 0-2 M lactose (Sigma, St. Louis, USA) to saturate the lectin binding sites and prohibit interaction with the sugars of tissue components. With order to rule out non-specific tissue binding, controls that had not previously been exposed to the biotinylated lectins were also incubated in Extravidin-HRP. The end outcome of all these processes was the total absence of any histochemical staining.

## Statistical Analysis:

The samples were analyzed in triplicates, and the results are presented as Means ± SD. Statistical analyses were done by using one way ANOVA and followed student t-test. Statistically the p-value of <0.05* was considered significant, whereas p values of <0.05* and <0.01** were considered very significant.

## Results:

**Figure 1:**
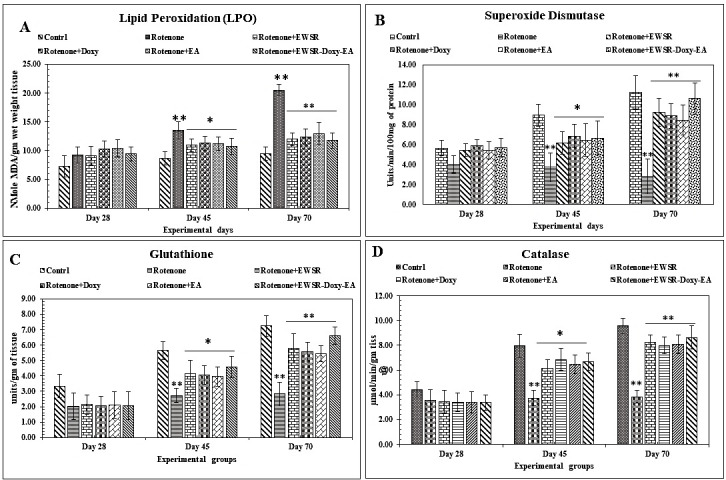
Influence of Rotenone exposure on Lipid peroxidation (LPO), Superoxide dismutase (SOD), Catalase (CAT), and Glutathione (GSH) rat brain at 28, 45 and 70 day in absence and presence neuroprotectants

In our earlier publications (under publication review), we have reported about the ROT influence on cytokine expression (RT-PCR, and ELISA), cytotoxicity (in vitro studies) and ROT induced neurochemical and behavioral altercations in the male Wistar rat brains. The concomitant treatment with neuroprotectants showed less cytotoxicity (*in vitro*), and administration of neuroprotectants to rats post ROT treatment ameliorated ROT induced alterations in time-dependent manner. ROT induction for 4 weeks has resulted in increased LPO in Grp II to VI when compared to the Grp I rats. Grp III to VI showed decreased levels of LPO in comparison to Grp II indicating anti-lipo-peroxidant activity of EWSR and other neuroprotectants individually and in combination (*p<0.05, **p<0.01), which was in time-dependent manner. Decreased anti-oxidant levels (SOD, CAT, and GSH) (Figure 1) were observed in the brains of Grp II to VI, compared to Grp I designate to ROT induced the oxidative stress. The administration of EWSR and other neuroprotectants individually and in combination (Grp III to VI) demonstrated the antioxidant property against ROT-induced toxicity with extended time of administration. The combination neuroprotectants has shown better ameliorating effects against ROT-induced toxicity (*p<0.05, **p<0.01).

## Histological findings:

## Hematoxylin and Eosin staining:

The tissue sections were all stained with hematoxylin and eosin (H and E) stain, and the image magnification is 40X. Pyramidal neurons in the Cornu Ammonis (CA1 and CA2) areas of the hippocampus have undergone morphological alterations as a result of ROT exposure, and there have also been neuronal losses, confirming ROT's toxicity. ROT-induced neuron death and morphological change have been minimised when EWSR has been administered post ROT administration. With time and continuous exposure, the toxic effects of the EWSR have been effectively mitigated in a time-dependent way, and the neurons now appear to be normal and similar to those of the controls. The number of neurons decreased, their shape changed and appeared to necrotic due chronic exposure to ROT. The ability of EWSR to protect neurons from ROT induced damage was demonstrated by the protecting the neurons morphology that was comparable to the controls. Neuron with normal morphology was seen in the rats receiving neuroprotectants after rotenone exposure, which demonstrated that the number of neurons increased steadily with EWSR administration alone and in combination.

The tissue sections were all stained with hematoxylin and eosin (H and E) stain, and the image magnification is 40X. Substantia nigral neurons in the mid brain showed morphological alterations as a result of ROT exposure, and there have also been neuronal losses, fibrosis, and infiltration of inflammatory cells confirming ROT's toxicity. ROT-induced neuron death and morphological change have been ameliorated when EWSR has been administered post ROT treatment alone and in combination with known drugs. With continuous administration of neuroprotectants such as EWSR, Doxy, EA and combination post ROT treatment were able to mitigate the ROT neurotoxicity, and protected the neurons from degenerating, controlled neuron morphology and appeared similar to the neurons in the control tissues. The above Figure 3 (Brain sections collected day 70) shows A). Control: Neurons showing normal morphology, B). Rotenone: Marked degenerative changes, loss of cellular detains, neuronal swelling and increase in neuropil, increase in inter neuronal space, C). ROT-EWSR: moderate focal inflammation, mild fibrous degeneration and pleiomorphic neurons, D). ROT-Doxy: moderate vacuolation and congestion with inflammation, E). ROT-EA: Moderate infiltration of inflammatory cells, neuronal degeneration and swekking, F). ROT-Combi: mild fibrosis and degenerative changes.

The Mean±SD values of six (n=6) individual observations; One way ANOVA (*p<0.05, **p<0.01, ***p<0.001 statistically significant). (MDA content expressed as in nM of MDA/gm wet weight of tissue, The SOD, CAT, GSH activities were expressed in units/min/g of protein.)

The immunohistopathological observations in the aforementioned figure demonstrate how microglial cells get activated and undergo morphological change when exposed to ROT (ramified to an amoeboid structure of microglia). The resident scavenger cells in the brain, called microglial cells, assist in clearing the debris of wounded and dying cells. However, due to mitotoxicity brought on by ROT, these resident cells get activated and go through morphological changes, which cause phagocytosis of both the injured and healthy neurons. Microglia activation is a sign of excitotoxicity and inflammation. EWSR and other neuroprotectants were administered post ROT treatment for next 40 days showed signs of amelioration and neuroprotection by turning active microglia into normal or ramified microglia (i.e Amoeboid form (AM) to less pathogenic ramified form (RM)).The active or amoeboid form microglia are darkly stained, whilst the less active or inactive microglia accept less stain and seem dull or ramified. This is how the DAB (3,3'-Diaminobenzidine) staining distinguishes between active and inactive microglia. Based on the observation, it is clear that EWSR and other neuroprotectants have anti-inflammatory properties that increase with extended administration time.

## Discussion:

**Figure 2:**
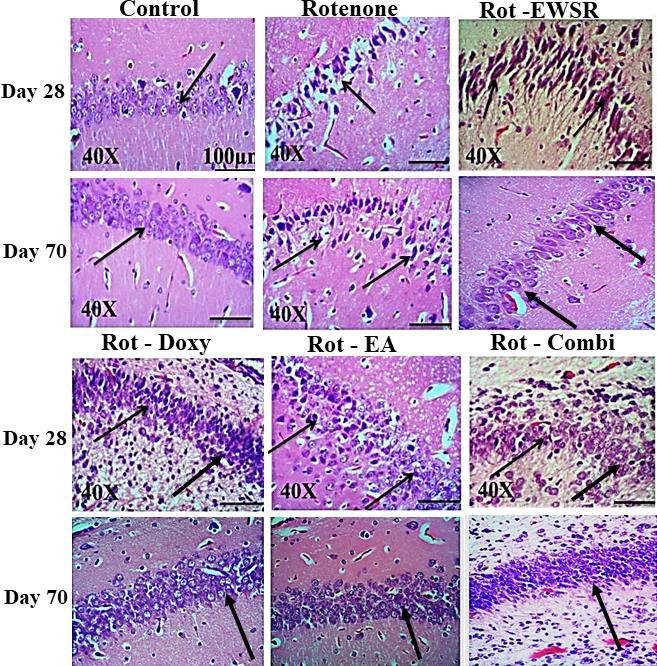
Rotenone-induced histopathological alterations in the hippocampus region of the brain in absence and presence neuroprotectants.

**Figure 3:**
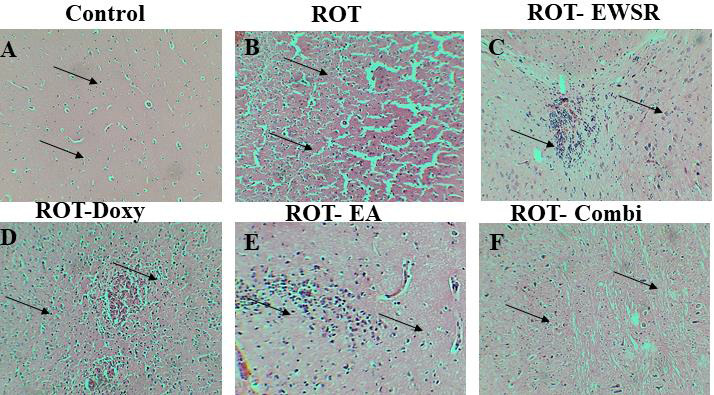
Rotenone-induced histopathological alterations in the midbrain region of the brain in absence and presence neuroprotectants.

**Figure 4:**
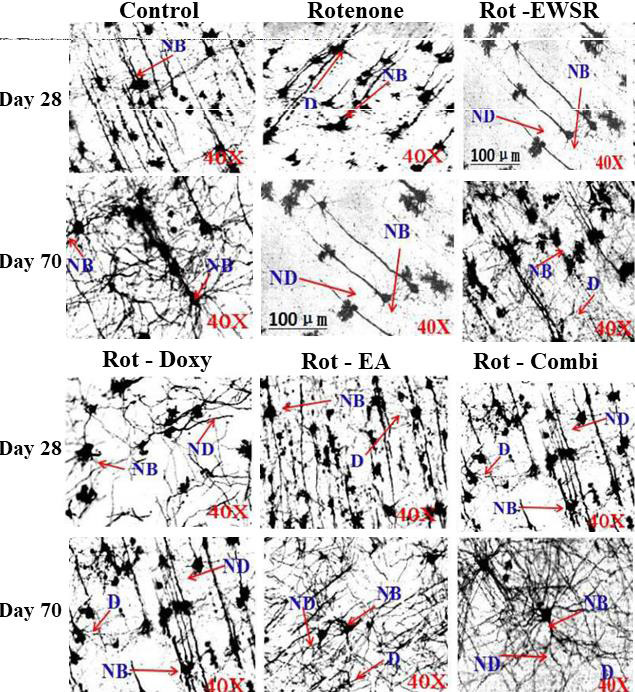
Rotenone-induced neurological alterations in the cortex region of brain in absence and presence EWSR and other protectants Rat brains were collected on the 28th and 70th days of the experiment. Golgi-Cox staining was used to examine the morphology, density, dendrite-axon interaction, and degeneration of neural cells in the cerebral cortex of the control, Rotenone, and Rotenone with neurprotectants groups. There is a 40X magnification. Dendrite (D), neuronal dendrite (ND), and neural bulb (NB). As seen in the above image, the experimental models (ROT, ROT-WSER, ROT-Doxy, ROT-EA, and ROT- Combi) exhibit morphological alterations, a decrease in the density of neural cells, loss of neurodendrites, and loss of axons as compared to control rats on day 28th. During the exposure time, the morphology of the neurons changed, the number of neurons dropped, and the axons, neurodendrites, and dendrites of the neurons were lost. Neurons with intact axons and dendrites were observed, and the morphology of neurons was similar to that of controls, indicating the neuroprotective ability of EWSR and other known neuroprotectants individually and in combination. In contrast to the rats receiving neuroprotectants post rotenone exposure, which showed the number of neurons increasing gradually with age and time of neuroprotectants administration, neurons with intact axons and dendrites were observed.

**Figure 5:**
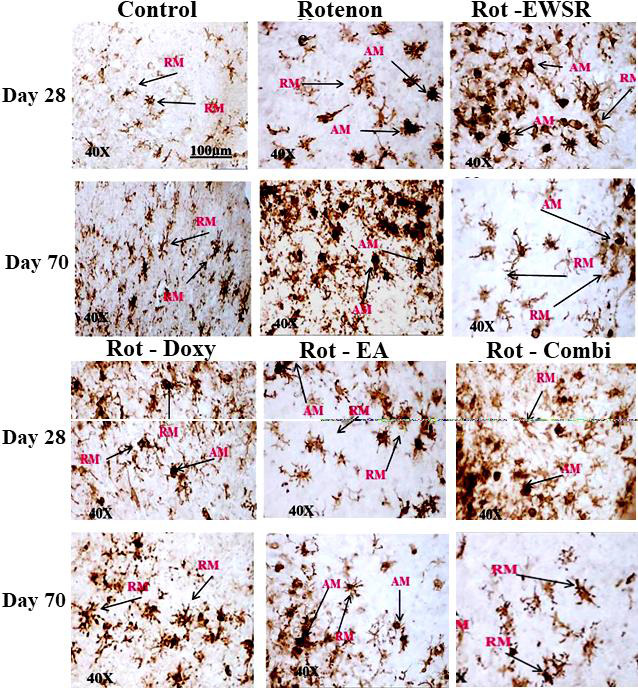
Microglial activation in the Mid brain region of rat on Rotenone exposure in presence and absence of neuroprotectants.

Rotenone (ROT) is known to induce many pathophysiological conditions in animal models, which pose several health problems as mentioned earlier. One of the main causes for the pathophysiological manifestations is suspected to be the involvement of free radical generations [27]. There are many reports about ROT-induced oxidative damage on the cellular membranes due to free radical generation [7].Oxidants and antioxidants hold a key in keeping the balance between the generation of free radicals and antioxidant systems present in the animal body. The oxidative damage is more whenever there is an exhaustion of dietary antioxidants or precursors of antioxidants [28]. One therapeutic approach against neurodegeneration is through the restoration of oxidative buffering capacity [5, 8]. The brain is especially susceptible to oxidative damage. In spite of the high rate of ROS production, the brain has a relatively low antioxidant defense system attributed to a high rate of oxidative metabolism and abundance of polyunsaturated fatty acids in the cell membrane [1, 29]. The elevated lipid peroxidation was observed in all experimental time periods in relation to the prolonged ROT ingestion in the hippocampus of the brain compared to control (Figure 2a) oxidative destruction of polyunsaturated fatty acids (PUFAs) of membrane phospholipids is a phenomenon generally termed lipid peroxidation. The LPO levels were less in EWSR and neuroprotectants treated groups when compared to ROT groups indicating the efficacy of the extract in reducing the ROT toxicity [10, 20]. Different ROS scavengers, the GSH dependent system is great importance including two antioxidants (SOD and GPx) and one intracellular antioxidant (GSH). The ROT accumulation occurs more in specific regions of brain observed [3]. The hippocampus showed a high accumulation of ROT than the cerebral cortex. The brain receives blood through the carotid arteries. Uninterrupted oxygen supply required for brain hence brain received abundant blood supply than other organs [28]. SOD is an important defense enzyme, which converts superoxide radicals (O2¯) to hydrogen peroxide [30]. There is ample evidence that SOD is important in protection against oxidation and lipid peroxidation. A decrease in the activity of free radical scavenging enzymes like SOD has been reported to occur in people living in areas of endemic rotenone [31]. Decreased SOD activity in Brain, liver, heart, and kidney of mice was reported in rats treated with ROT3. One probable mechanism by which ROT might affect the SOD activity is that at high concentration ROT is likely to inhibit superoxide dismutase resulting in the accumulation of large amounts of free radicals and peroxide causing cell damage [27]. ROT intoxication with the dosage of 2mg/kg.bw has resulted in the depletion of antioxidants such as GST, GSH, and CAT, in all the regions of the brain may be due to the over-utilization of glutathione in the reduction of free radical-induced stress44. The expression and increased activity of antioxidant enzymes were observed in the tissues which are subjected to oxidative stress, and they ameliorate the damage caused by free radicals [26]. Sharma *et al*. [32] has reported that just the enhanced activity of the antioxidant enzymes is not enough to control the impending damage in many conditions of oxidative stress. The observation made in the present study does corroborate with the results published by Saravanan *et al*. [33], and Prakash *et al*. [34]. Decreased SOD, CAT, GSH activity and diminished Vit A levels was observed in Grp II when compared to the Grp I, III and VI. Grp III and VI showed elevated levels of antioxidants in comparison to the Grp II, indicating the anti-oxidative property of EWSR and neuroprotectants and it was also observed that the efficacy of EWSR and neuroprotectants was dose responsive (Figure 1). The simultaneous administration of EWSR and neuroprotectants has successfully restored the levels of antioxidants (GSH, CAT, and SOD) and helped in reducing the ROT-induced neurodegeneration in the brain, which could be attributed to treatment with anti-oxidant rich EWSR and neuroprotectants alone and in combination (Figure 1). The SNPc is the prominent affected region with the ROT than the hippocampus and cerebral cortex [4]. In the present study the biochemical parameters (Oxidative markers) in the brain correlated with the histopathological studies demonstrating all SNPc and hippocampal regions being affected by the ROT toxicity[35].Neuronal morphology alterations in pyramidal and dopaminergic neurons in hippocampus and SNpc observed in ROT group sections compared to the control group (Figure 2 and Figure 3). Golgi-Cox staining has helped in observing the ROT-induced alterations in the morphology of neural cells in the cerebral cortex, the neurons appeared degenerated and lost their axons and dendrites. The co-treatment of EWSR and neuroprotectants to the ROT treated postnatal rats, has shown normal neuronal morphology and the neurons appeared healthy and had intact axons and dendrites, indicating the protective effects of EWSR and neuroprotectants treatment against ROT induced neurotoxicity and better amelioration was observed to be dose-dependent (Figure 4). As EWSR and neuroprotectants is a rich source of Alkaloids, Zinc, Copper, etc. which are the key factors in maintaining neuronal functioning [36].A rotenone-induced animal model of PD exhibits microglial activation, which is accompanied by limited astrocytosis [37]. In elderly rats, glial cell activation is more severe. A widespread increase in astrocytes and microglia is caused by unilateral rotenone infusion into the medial forebrain bundle, excluding the SN [32]. The microglial activation was observed in ROT intoxicated brains of the rat and the number of activated microglia (Figure 5) were increasing with dose duration and age. The EWSR and neuroprotectants administration along with ROT, showed reduced microglial activation in the whole brain, indicating the efficiency of EWSR and neuroprotectants in controlling the microglia activation, generation of free radicals (oxygen and nitrogen) and production of pro-inflammatory markers such as inducible nitrogen synthase (iNOS) etc. [37, 38].

## Conclusion:

ROT exposure altered the profile of antioxidants, neuronal morphology in rats. Elevated LPO levels were observed in ROT-exposed rats with increasing age and duration when compared to the controls and EWSR AND NEUROPROTECTANTS treated. The levels of antioxidants such as SOD, CAT, and GSH levels drastically got reduced in ROT-treated rats, whereas the levels were near to control in EWSR and neuroprotectants administered rats. Rats treated with EWSR and other neuroprotectants post ROT treatment has shown similar neuron morphology to that of the controls. The anti-lipoperoxidative, anti-oxidative and anti-inflammatory property of EWSR and neuroprotectants have shown ameliorative effects in brain indicated by biochemical and histo-morphological studies performed, thus it certainly proves EWSR is neuroprotective in nature due to its nutrient rich composition.
